# Occurrence and molecular characterization of *Giardia duodenalis* in domestic and wild mesocarnivores in Bosnia and Herzegovina

**DOI:** 10.1016/j.ijppaw.2026.101197

**Published:** 2026-01-22

**Authors:** Azra Bačić, Naida Kapo, Jasmin Omeragić, Šejla Goletić Imamović, Toni Eterović, Ilma Terzić, Adis Softić, Vedad Škapur, Teufik Goletić

**Affiliations:** a University of Sarajevo - Veterinary Faculty, Sarajevo, Bosnia and Herzegovina; b University of Sarajevo - Faculty of Agriculture and Food Science, Sarajevo, Bosnia and Herzegovina

**Keywords:** *Giardia duodenalis*, Molecular epidemiology, Mesocarnivores, Zoonotic assemblages, Wildlife-domestic interface, Bosnia and Herzegovina

## Abstract

*Giardia duodenalis* is a protozoan parasite with worldwide distribution and recognized zoonotic potential. Data on its molecular epidemiology in Bosnia and Herzegovina (BiH) are scarce, particularly in wild mesocarnivores. This study aimed to investigate the occurrence and genetic characterization of *G. duodenalis* in domestic and wild mesocarnivores across BiH. A total of 520 fecal samples were collected between 2023 and 2025, including dogs (*Canis lupus familiaris*, n = 433), cats (*Felis catus*, n = 21), red foxes (*Vulpes vulpes*, n = 39), golden jackals (*Canis aureus*, n = 17), European pine martens (*Martes martes*, n = 5), grey wolves (*Canis lupus*, n = 1), European badgers (*Meles meles*, n = 2), and European wildcats (*Felis silvestris*, n = 1). Screening was performed using fecal flotation and immunofluorescence assay (IFAT), with selected samples further analyzed by high-resolution melting (HRM) real-time PCR (qPCR-HRM) and targeted next-generation sequencing (NGS).

Overall, *G. duodenalis* was detected in 20.96 % (109/520) of samples by flotation and IFAT. Cats showed the highest positivity rate (71.43 %), followed by dogs (21.02 %), whereas wild mesocarnivores exhibited substantially lower detection rates (5.13 % in red foxes and 5.88 % in golden jackals). Among dog subpopulations, hunting dogs showed the highest positivity (49.52 %) compared with shelter dogs (6.72 %). Molecular typing revealed assemblage D as predominant (65.91 %), followed by assemblages B (18.18 %), C (6.82 %), and F (4.55 %), with occasional mixed profiles. Assemblage D occurred across multiple hosts, while the zoonotic assemblage B was detected exclusively in wild canids.

This study provides the first molecular epidemiological evidence of *G. duodenalis* assemblage circulation among domestic and wild mesocarnivores in Bosnia and Herzegovina. The findings identify cats and hunting dogs as key hosts contributing to parasite circulation and demonstrate limited but epidemiologically meaningful involvement of wild mesocarnivores, underscoring the importance of integrated One Health surveillance to assess transmission risks at the domestic-wildlife-human interface.

## Highlights

• *Giardia duodenalis* detected in 20.96 % of mesocarnivores in Bosnia and Herzegovina.
• Assemblage D dominated domestic hosts; assemblage B occurred only in wildlife.
• Molecular screening identified subclinical and low-intensity infections in wildlife.

## Graphical abstract

**Figure:**
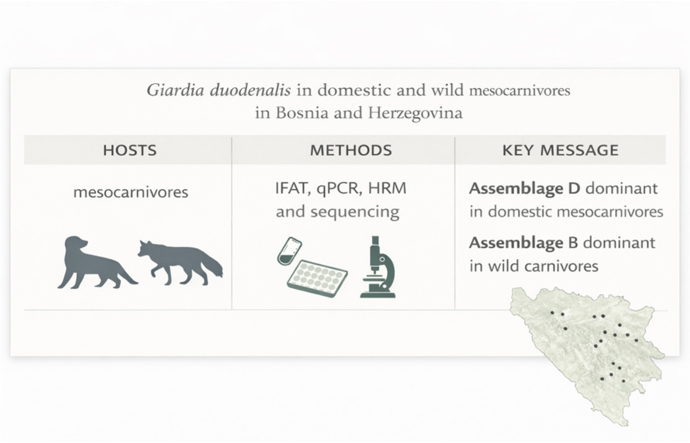

## 1 Introduction

*Giardia duodenalis* is an intestinal flagellated protozoan capable of causing mild to severe gastrointestinal disease in a wide range of domestic and wild species (Feng and Xiao, 2011). *Because several assemblages circulate simultaneously among humans, livestock, pets, and wildlife, G. duodenalis represents a pathogen of clear One Health relevance.* The species complex comprises eight genetic assemblages (A–H) (Ryan and Cacciò, 2013; Heyworth, 2016). Assemblages A and B occur in humans, and various domestic and wild species, while C and D predominantly infect dogs and wild canids, E hoofed animals, and F, G, and H felines, rodents, and marine mammals, respectively (Thompson, 2004; Feng and Xiao, 2011; Ryan and Cacciò, 2013). Only assemblages A and B are recognized human pathogens (Feng and Xiao, 2011), while zoonotic involvement of other assemblages appears limited and mostly linked to non-productive human infections (Gelanew et al., 2007; Foronda et al., 2008; Traub et al., 2009). Globally, giardiasis is one of the most common intestinal protozoa, with human prevalence estimates varying from 2–5 % in industrialized countries to 30–40 % in developing countries (Stephen et al., 2025). Reflecting its significant public health impact, *Giardia* spp. is ranked as the seventh most important foodborne parasite in Europe (Bouwknegt et al., 2018).

In domestic carnivores, particularly dogs and cats, *G. duodenalis* is among the most frequently detected intestinal protozoa, and may result in either asymptomatic carriage or gastrointestinal disease (Ballweber et al., 2010). Mesocarnivores are particularly relevant from a One Health perspective because many species exhibit high ecological plasticity, exploit human-modified environments, and readily traverse the domestic–wildlife interface. Ecologically, mesocarnivores are species whose diet consists of roughly 50–70 % animal-derived prey, placing them between hypocarnivores and hypercarnivores within established carnivore trophic guilds (Roemer et al., 2009; Ritchie and Johnson, 2009; Kays et al., 2015). Their foraging flexibility, frequent scavenging behavior, and overlap with peri-urban habitats increase opportunities for environmental contamination and multi-host circulation of both host-adapted and zoonotic *Giardia duodenalis* assemblages. Within this framework, the inclusion of grey wolves, although trophically classified as hypercarnivores, allows extension of the analysis along a carnivore ecological continuum, capturing pathogen circulation across species differing in diet but sharing exposure pathways and landscape connectivity. Consistent with this perspective, although studies in wildlife remain limited due to logistical challenges, molecular surveys have shown that wildlife can harbor a wide range of assemblages, underscoring their potential role in local transmission networks (Beck et al., 2011; Debenham et al., 2017; Figueiredo et al., 2023).

At the regional level, available data on *G. duodenalis* in mesocarnivores remain fragmented, and Bosnia and Herzegovina in no exception. Previous studies using fecal flotation and immunofluorescence detected infections in 6.54 % of pet dogs and 24.76 % of stray dogs (Omeragić et al., 2021), while the only survey in red foxes reported a prevalence of 7.32 % (Hodžić et al., 2014). However, these studies focused solely on occurrence and did not include molecular characterization. As a result, assemblage-level distribution across domestic and wild mesocarnivores remains unknown, and local transmission dynamics, including potential contact points between wildlife, free-roaming carnivores, and humans, remains poorly defined.

To address this knowledge gap, we investigated the occurrence and molecular assemblage distribution of *G. duodenalis* in domestic and wild mesocarnivores in Bosnia and Herzegovina. Therefore, the aim of this study was to investigate the occurrence and molecular assemblage distribution of *G. duodenalis* in domestic and wild mesocarnivores in Bosnia and Herzegovina. We hypothesized that these hosts predominantly harbor host-adapted assemblages, with occasional detection of zoonotic assemblages reflecting increased overlap of wildlife, free-roaming carnivores, and human-modified environments. By generating the first multi-host molecular dataset for the country, this study provides essential baseline information for clarifying the role of mesocarnivores in local transmission cycles and contributes to a better understanding of potential cross-species and zoonotic interfaces within a One Health context.

## 2 Materials and methods

### 2.1 Study area and materials

**Fig. 1:**
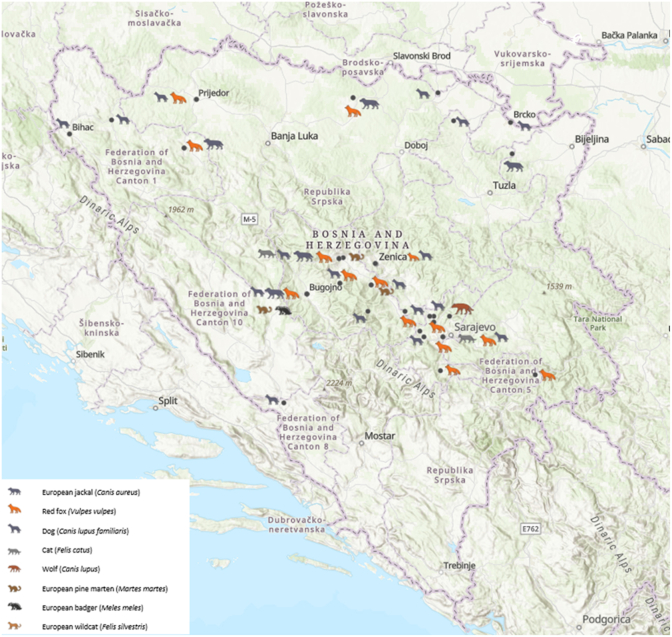
Locations of the 22 municipalities in Bosnia and Herzegovina included in the sampling of domestic and wild mesocarnivores. Map created using ArcGIS® Online (ESRI, Redlands, CA, USA).

Between 2023 and 2025, a total of 520 fresh fecal samples were collected from domestic and wild mesocarnivores across 47 geolocations within 22 municipalities in Bosnia and Herzegovina (Fig. 1). Sampling was performed in collaboration with local veterinary practices, wildlife management units and hunters’ associations. The final dataset included samples from dogs (*Canis lupus familiaris*, n = 433), cats (*Felis catus*, n = 21), red foxes (*Vulpes vulpes*, n = 39), golden jackals (*Canis aureus*, n = 17), European pine martens (*Martes martes*, n = 5), wolves (*Canis lupus*, n = 1), European badgers (*Meles meles*, n = 2), and European wildcats (*Felis silvestris*, n = 1).

Fecal samples from dogs and cats were collected from both healthy and clinically affected animals presented to practicing veterinarians, including individually owned pets, hunting dogs, and animals kept in shelters or kennels. Owners of pet and hunting dogs were contacted directly, invited to participate in the study, and asked to complete a standardized epidemiological questionnaire. Samples from wild animals were collected post-mortem, directly from the rectum during necropsies of legally hunted or road-kill animals, in accordance with national wildlife management regulations. No animal was killed specifically for the purposes of this study.

All samples were transported under cold chain (4–8 °C) to the diagnostic laboratories of University of Sarajevo - Veterinary Faculty and processed within two days of collection.

### 2.2 Fecal flotation and IFAT assay

Fecal samples were examined using zinc sulfate flotation (specific gravity 1.18) (Zajac and Conboy, 2012) and indirect immunofluorescence test (MERIFLUOR® *Cryptosporidium/Giardia* test, Meridian Bioscience, Inc.) following previously described protocols (Johnston et al., 2003). A fecal sample was considered positive only when *Giardia* cysts were detected by both fecal flotation and IFAT. All parasitological analyses were conducted at the Laboratory of Parasitology, University of Sarajevo - Veterinary Faculty. One fecal sample per animal was processed.

### 2.3 Molecular methods

Molecular analyses were performed at the Laboratory for Molecular Genetics and Forensic Investigations, University of Sarajevo – Veterinary Faculty. qPCR-HRM analyses were applied to all parasitologically positive samples obtained from domestic mesocarnivores (n = 106), whereas all samples originating from wild mesocarnivores (n = 66), irrespective of parasitological status, were subjected to molecular testing.

Molecular testing followed the general workflow described by Bahramdoost et al. (2021), with methodological adaptations related to reagent composition and analytical platform. Specifically, amplification of the targeted *gdh* fragment of *Giardia duodenalis* was performed using an alternative qPCR master mix, and assays were run on a different real-time PCR platform. qPCR-HRM analyses were conducted using the Mic qPCR Cycler (BioMolecular Systems, Australia), with subsequent data processing performed in micPCR software version 2.8.13 (BioMolecular Systems, Upper Coomera, Australia). Melting curves were generated automatically, and corresponding melting temperature (Tm) values were recorded.

Prior to molecular testing, fecal material was prepared for DNA extraction. Approximately 200 mg of each fecal sample was transferred into a sterile 1.5 mL microcentrifuge tube. To facilitate cell lysis and ensure adequate homogenization, 1.4 mL of ASL buffer (QIAamp DNA Stool Mini Kit, Qiagen, Hilden, Germany) was added, followed by vigorous vortexing until a uniform suspension was obtained. Samples were subsequently heated at 95 °C for 5 min to enhance disruption of microbial cells. DNA extraction was then performed using the QIAamp DNA Stool Mini Kit (Qiagen, Hilden, Germany) according to the manufacturer's instructions, with minor modifications. Specifically, DNA was eluted in two sequential steps—first with 70 μL of buffer ATE and subsequently with 70 μL of PCR-grade water—to reduce potential interference of elution buffer components with downstream NGS-based analyses.

#### 2.3.1 qPCR setup with HRM analysis (qPCR-HRM)

qPCR reactions were prepared in a final volume of 20 μL, comprising 10 μL of Forget-Me-Not™ EvaGreen® qPCR Master Mix (Low ROX) (Biotium, USA), 0.25 μM of each primer (FHRM and RHRM), and 3 μL of template DNA. The remaining volume was adjusted with RNase-free water. Amplification was performed using the following thermal profile: an initial enzyme activation step at 95 °C for 2.5 min, followed by 40 cycles of denaturation at 95 °C for 30 s, annealing at 61 °C for 35 s, and extension at 72 °C for 35 s.

Upon completion of amplification, high-resolution melting (HRM) analysis was conducted. This included an initial stabilization step at 95 °C for 10 s and 72 °C for 40 s, followed by a continuous melting ramp from 73 °C to 92 °C at a rate of 0.025 °C·s^−1^. Fluorescence data (FAM channel) were acquired using the BMS Workbench software (version 1.4.10) for melting profile analysis.

### 2.4 Next-generation sequencing of selected *gdh* amplicons

Next-generation sequencing (NGS) was performed on selected samples that met predefined inclusion criteria. These comprised, first, aberrant or ambiguous melting temperatures (Tm) obtained by qPCR-HRM, indicative of atypical melting profiles warranting sequence-level resolution, and second, Ct values within a range (approximately 26–28) compatible with the generation of sufficient amplicon quantity for downstream sequencing. To confirm PCR-based molecular findings and resolve samples with discordant parasitological and molecular screening results, NGS was applied to four selected *gdh* amplicons originating from both domestic and wild mesocarnivores.

Sequencing was performed on four selected *gdh* amplicons originating from both domestic and wild mesocarnivores (sample 76A - red fox; 113A - golden jackal; 121A - hunting dog; 142A - red fox). These four amplicons were selected based on predefined quality criteria (amplicon yield) and the need to resolve atypical/ambiguous HRM patterns; they were not intended to represent all host species or assemblages.

Amplicons of the *gdh* gene generated by HRM analysis were purified using AMPure XP beads (Beckman Coulter, Brea, USA) and quantified using the Qubit™ 1X dsDNA High Sensitivity Assay Kit (Thermo Fisher Scientific, Waltham, USA) on a Qubit 4 Fluorometer, according to the manufacturer's instructions. Sequencing libraries were prepared using the Native Barcoding Kit 24 V14 (SQK-NBD114.24; Oxford Nanopore Technologies, Oxford, UK) and sequenced on an R10.4.1 flow cell using the Oxford Nanopore MinION Mk1C platform.

Raw FAST5 files were basecalled in real time using high-accuracy basecalling with a minimum quality score of 10 in MinKNOW software (version 20.10.3). Sequencing was run for 10 h to ensure sufficient read depth and coverage. Sequence read quality was additionally assessed using Seqkit (version 2.8.2; https://bioinf.shenwei.me/seqkit/). Reads were mapped against reference *gdh* sequences representing all known *Giardia duodenalis* genetic assemblages (GenBank accession numbers: DQ100288 [A], EF507598 [AI], EF507674 [AII], JN204453 [B], AY178749 [BI], KP026305 [BIII], KJ741295 [C], KY753403 [D], OR455159 [E], LC341552 [F], and GU176101 [G]) using minimap2 (version 2.30; https://github.com/lh3/minimap2). Mapped reads were visually inspected using UGENE (version 52.0), and final consensus sequences were generated with iVar (version 1.4.3; https://github.com/andersen-lab/ivar). Genetic assemblages were assigned by comparison of consensus sequences using the NCBI BLAST tool.

### 2.5 Statistical analysis

Differences in parasitological detection rates between host categories and dog subcategories were assessed using the chi-square test. Where appropriate, post-hoc Z-tests for equality of proportions were applied with Bonferroni correction. Statistical significance was set at p < 0.05. Analyses were performed using Stata 15/SE (StataCorp, College Station, TX, USA).

## 3 Results

### 3.1 Fecal flotation and IFAT assay

The overall detection rate of *G. duodenalis* in the examined mesocarnivores was 20.96 % (109/520) (Table 1). Among domestic hosts, the parasite was detected in 21.02 % (91/433) of samples originated from dogs, while in cats the detection rate of *G. duodenalis* was 71.43 % (15/21). Among wild species, *G. duodenalis* was detected in 5.13 % (2/39) of red foxes, 5.88 % (1/17) of golden jackals. Pine martens (n = 5), Grey wolf (n = 1), European badgers (n = 3) and European wildcat (n = 1) were tested negative for *G. duodenalis.* The detection rate of *G. duodenalis* varied significantly among dog categories (χ^2^ = 92.30; df = 3; p < 0.001). Hunting dogs exhibited the highest proportion of positive samples (49.52 %), followed by pet dogs (38.10 %) and breeding dogs (27.78 %). The detection rate in sheltered dogs was 6.72 %. Post-hoc comparisons performed using Z-tests for equality of proportions with Bonferroni correction, revealed no significant differences among hunting, pet, and breeding dogs (p > 0.05), whereas sheltered dogs differed significantly from all other groups (p < 0.05).

**Table 1:** The detection rate of *G. duodenalis* in domestic and wild mesocarnivores in Bosnia and Herzegovina using fecal flotation and IFAT test.

Host species	n	n_pos_	%	95 % CI	p-value
**Dog (*Canis lupus familiaris*)**	433	91	21.02	17.44–25.15	–
**Dog subcategories (totals included above)**					
Sheltered dog	268	18	6.72^a^	4.29–10.37	<0.001
Hunting dog	105	52	49.52^b^	40.15–58.93	–
Pet dog	42	16	38.10^b^	25.0–53.19	–
Breeding dog	18	5	27.78^b^	12.5–50.87	–
**Cat (*Felis catus*)**	21	15	71.43	50.04–86.19	–
**Red fox (*Vulpes vulpes*)**	39	2	5.13	1.42–16.89	–
**Golden jackal (*Canis aureus*)**	17	1	5.88	1.05–26.98	–
**Pine marten (*Martes martes*)**	5	0	0	–	–
**Grey wolf (*Canis lupus*)**	1	0	0	–	–
**European badger (*Meles meles*)**	3	0	0	–	–
**European wildcat (*Felis silvestris*)**	1	0	0	0.00–95.00	–

**TOTAL**	520	109	20.96	17.68–24.67 %	–

### 3.2 qPCR-HRM

Molecular testing using qPCR-HRM analysis identified *G. duodenalis* DNA in 43.02 % (74/172) of examined mesocarnivore samples (Table 2). Among domestic hosts, dogs showed a detection rate of 36.26 % (33/91), with positive samples obtained across all dog categories. Hunting dogs exhibited the highest proportion of qPCR-positive results (42.31 %), followed by pet dogs (43.75 %), breeding dogs (20.00 %), and sheltered dogs (16.67 %). In cats, the molecular detection rate was 66.67 % (10/15). Among wild hosts, *G. duodenalis* was detected in 43.59 % (17/39) of red foxes and 58.82 % (10/17) of golden jackals. Lower detection rates were observed in pine martens (40.00 %; 2/5) and grey wolf (1/1). European wildcat (1/1) was also positive, while European badgers tested negative (Table 2.).

**Table 2:** qPCR-HRM results of *G. duodenalis* in domestic and wild mesocarnivores in Bosnia and Herzegovina.

Host species	n	n_pos_	%	95 % CI	Assemblages genotyped
**Dog (*Canis lupus familiaris*)**	91	33	36.26	27.13–46.51	C(2); D(17); F(2); F + C(1)
**Dog subcategories *(included above)***					
Sheltered dog	18	3	16.67	5.84–39.22	D(2); F(1)
Hunting dog	52	22	42.31	29.87–55.81	D(13); C(2); F(1); F + C(1)
Pet dog	16	7	43.75	23.10–66.82	D(2)
Breeding dog	5	1	20.00	3.62–62.45	–
**Cat (*Felis catus*)**	15	10	66.67	41.71–84.82	D(3)
**Red fox (*Vulpes vulpes*)**	39	17	43.59	29.30–59.02	B(6); D(4)
**Golden jackal (*Canis aureus*)**	17	10	58.82	36.01–78.39	B(1); C(1); D(2); C + D(1)
**Pine marten (*Martes martes*)**	5	2	40.00	11.76–76.93	D(2)
**Grey wolf (*Canis lupus*)**	1	1	100.00	20.65–100	B(1)
**European badger (*Meles meles*)**	3	0	0.00	0–56.15	–
**European wildcat (*Felis silvestris*)**	1	1	100.00	20.65–100	D(1)

**TOTAL**	**172**	**74**	**43.02**	**35.80**–**50.40**	**44**

Assemblage assignment was obtained for 44 isolates, while two additional isolates from jackals could not be genotyped due to insufficient sequence resolution. For the remaining qPCR-positive samples (n = 28), assemblage determination was not reported because genotyping quality criteria were not met (e.g., ambiguous HRM profiles and/or insufficient amplicon quality/quantity for reliable sequencing-based confirmation). Among genotyped samples, assemblage D was the most frequently detected (29/44; 65.91 %), followed by assemblage B (8/44; 18.18 %), assemblage C (3/44; 6.82 %), and assemblage F (2/44; 2.27 %). Mixed assemblage profiles (F + C and C + D) were each recorded in one sample each (2.27 %). Assemblage distribution varied among host species. Dogs (n = 22) carried assemblages C, D, F, and one mixed F + C profile. Foxes (n = 10) harbored assemblages B and D, whereas jackals (n = 5) carried assemblages B, C, D, and one mixed C + D profile, in addition to the two non-typable isolates. Wolf harbored zoonotic B genotype. Cats (n = 3), martens (n = 2), and the wildcat (n = 1) were all positive exclusively for assemblage D (Table 2).

### 3.3 Next-generation sequencing (NGS) of selected *gdh* amplicons

Among the sequenced samples, Ct values ranged from 27.1 to 32.1, and DNA concentrations from 4.68 to 5.28 ng/μL. Consensus sequences aligned successfully with reference databases, enabling assignment to assemblage B (76A, 142A), assemblage C (113A), and a mixed F/C assemblage profile (121A). NGS outputs and genotype assignments are summarised in Table 3. All consensus sequences were deposited in GenBank under accession numbers PX237205, PX237209 (assemblage B), PX237206, PX237207 (assemblage C), PX237208 (assemblage F).

**Table 3:** NGS-based genotyping results of selected Giardia duodenalis *gdh* amplicons from domestic and wild mesocarnivores.

Sample ID	Host species	Sampling round	Ct value	Tm (°C)	DNA conc. (ng/μL)	Assemblage
76A	Fox	I	28.0	85.02	3.17	B
113A	Jackal	II	27.1	87.00	5.28	C
121A	Dog	I	32.1	81.32/87.5	3.60	F; C
142A	Fox	I	29.8	86.16	N/A	B

## 4 Discussion

This study provides the first comprehensive molecular epidemiological assessment of *G. duodenalis* in mesocarnivores in Bosnia and Herzegovina, expanding on earlier national surveys that relied exclusively on flotation or immunofluorescence without genotypic characterization or evaluation of zoonotic potential (Hodžić et al., 2014; Omeragić et al., 2015, 2021). By integrating parasitological and molecular approaches, we generated the first baseline dataset describing prevalence patterns and assemblage structure across domestic and wild hosts in the country. Domestic animals exhibited markedly higher positivity than wildlife, while molecular typing revealed a predominance of host-adapted assemblages (C, D, F) and limited circulation of the zoonotic assemblage B, highlighting complex transmission dynamics at the domestic-wildlife interface.

### 4.1 Domestic mesocarnivores

Domestic mesocarnivores demonstrated substantially higher *G. duodenalis* positivity than wild species, with marked heterogeneity among dog populations. Higher detection rates observed in hunting and privately-owned dogs compared with shelter dogs likely reflect differences in management practices, environmental exposure, and frequency of antiparasitic treatment. This pattern is consistent with European observations identifying husbandry, ecological factors, and diagnostic sensitivity as major determinants of apparent *G. duodenalis* prevalence (Lalle and Cacciò, 2023). Across Europe, reported prevalence in dogs varies widely, largely reflecting differences in sampling design, population structure, and laboratory protocols (Solarczyk et al., 2019; Uiterwijk et al., 2018). Molecular typing revealed a clear predominance of host-adapted assemblage D in dogs, followed by assemblages C and F and occasional mixed infections (F + C), in line with evidence that canids predominantly harbor assemblages C and D, while zoonotic assemblages A and B occur sporadically (Barutzki and Schaper, 2003; Agresti et al., 2022). The detection of assemblage F, typically associated with felids, suggests occasional host-barrier crossing, a phenomenon previously documented in other regions (Silva et al., 2022).

Cats exhibited a high positivity rate, underscoring their potential epidemiological relevance, although this finding should be interpreted cautiously given that most sampled individuals presented with gastrointestinal signs. European studies report substantial variability in feline prevalence depending on diagnostic approach and population type (Berrilli et al., 2004; Bouzid et al., 2015). The exclusive detection of assemblage D in cats contrasts with the more commonly reported dominance of assemblages A and F in Europe (Jaros et al., 2011) and suggests occasional spillover from sympatric canid populations, particularly in peri-urban settings where cats and dogs share habitats and exposure pathways. Similar cross-species transmission events have been reported in Romania, Germany and Japan (Gyӧrke et al., 2016; Sommer et al., 2018; Iijima et al., 2018). Overall, findings from domestic mesocarnivores highlight the complex interplay between host ecology, animal management practices, and environmental exposure in shaping *G. duodenalis* transmission dynamics.

### 4.2 Wild mesocarnivores

Wild mesocarnivores showed low overall *G. duodenalis* positivity when assessed by conventional parasitological methods, with infections detected primarily in red foxes and golden jackals. In contrast, molecular assays revealed a substantially broader occurrence across species, including pine martens, wolves, and wildcats, indicating that low-intensity or subclinical infections frequently escape detection by flotation and immunofluorescence alone. Accordingly, molecular prevalence should be interpreted as a reflection of diagnostic sensitivity rather than true infection pressure in wild populations. When conservative, parasitology-based estimates are considered, the prevalence observed in red foxes in Bosnia and Herzegovina (5.1 %) is slightly lower than that previously reported in the country (7.3 %) (Hodžić et al., 2014), but closely matches findings from neighboring Croatia (4.5 %) (Beck et al., 2011). Across Europe, reported prevalence in red foxes varies widely, ranging from 4.6 % to 27.4 % (Hamnes et al., 2007; Mateo et al., 2017; Figueiredo et al., 2023; Mateusa et al., 2024; Dwużnik-Szarek et al., 2025), a variability that likely reflects differences in sampling strategies, ecological conditions, and diagnostic approaches rather than true geographic contrasts in transmission intensity.

Assemblage patterns in red foxes (B and D) differed from those reported in Croatia, where assemblage A predominated (Beck et al., 2011), Norway and Romania, where assemblages A and B were most frequently detected (Hamnes et al., 2007), Sweden, where assemblage B predominated (Debenham et al., 2017), and Portugal, where assemblages C and D, including mixed C + D profiles, were reported (Figueiredo et al., 2023). These differences suggest regional structuring of *G. duodenalis* in European fox populations. Despite the overall low prevalence observed in Bosnia and Herzegovina, the detection of zoonotic assemblage B indicates a limited but epidemiologically relevant role in local transmission, while the recurrent identification of assemblage D (40 % in our series) supports the existence of a canid-adapted transmission cycle linking dogs, foxes, and golden jackals.

This study provides the first molecular evidence of *G. duodenalis* in golden jackals in Bosnia and Herzegovina. Golden jackals carried assemblages B, C, and D, including one mixed infection (C + D), consistent with the only previous European report from Croatia, which identified a mixed A + B infection in a single jackal (Beck et al., 2011). In the Jordan Basin, Israel, *G. duodenalis* was detected in 4.2 % of golden jackals, with molecular characterization revealing assemblage D (Arussi et al., 2024). Given the rapid demographic expansion of golden jackals across Europe and their increasing use of anthropogenically modified and peri-urban habitats, these findings highlight their potential role in facilitating parasite exchange at the domestic–wildlife interface. Published data on *G. duodenalis* in European grey wolves remain scarce. In Poland, *G. intestinalis* (syn. *G. duodenalis*) was detected in only 1.5 % of wolves, with the single genotyped isolate belonging to assemblage D. In contrast, in the present study, a wolf sample that was negative by flotation tested positive by qPCR-HRM analysis, with molecular typing revealing the zoonotic assemblage B, a genotype not commonly reported in wolves across Europe.

Data for other wild mesocarnivores in Europe are limited. Two of five pine martens examined in the present study carried assemblage D, whereas a beech marten in Spain could not be genotyped (Mateo et al., 2017). A single wildcat (*Felis silvestris*) examined here tested positive for *G. duodenalis* and was genotyped as assemblage D. Published European data for wildcats remain scarce, with a prevalence of 17.6 % reported by coprological methods in Croatia (Martinković et al., 2017) and a single PCR-positive case in Luxembourg typed as assemblage B (Solarczyk et al., 2019), indicating zoonotic potential.

### 4.3 Assemblage structure & zoonotic implications

Molecular typing revealed a dominance of host-adapted assemblages C and D, consistent with a stable, canid-associated transmission cycle within mesocarnivores in Bosnia and Herzegovina. In parallel, the detection of zoonotic assemblage B in wild hosts suggests that these populations may contribute to the environmental maintenance of lineages relevant to human infection. The absence of assemblage A likely reflects low regional prevalence rather than true absence and does not exclude its circulation at sub-detectable levels. The detection of atypical and mixed profiles, such as feline assemblage F in dogs and canid assemblage D in cats, highlights the ecological plasticity of *G. duodenalis* and occasional cross-species transmission, particularly in peri-urban settings where hosts share habitats and exposure pathways.

From a public health perspective, the presence of assemblage B points to potential environmental exposure risks for humans, even if mesocarnivores primarily function as secondary rather than primary sources. Overall, the observed assemblage pattern reflects the coexistence of host-adapted and zoonotic lineages and underscores the need for continued molecular surveillance at the domestic–wildlife interface.

### 4.4 Genetic diversity and HRM–NGS integration

The integration of HRM genotyping with nanopore-based sequencing revealed considerable genetic heterogeneity among *G. duodenalis* isolates, including mixed assemblage profiles. Minor deviations in melting temperatures observed among the analyzed isolates likely reflected underlying sequence polymorphisms, as previously reported within assemblages B, C, and D in European mesocarnivores. Reported Tm ranges for assemblages A, B, C, and D (Bahramdoost et al., 2021) were broadly consistent with the patterns observed in this study (Table 3), with minor shifts attributable to differences in reagent chemistry and qPCR platform performance. MinION sequencing confirmed that these deviations corresponded to genuine sequence polymorphisms. Notably, sample 121A exhibited a dual melting-peak profile and multiple haplotypes, underscoring the capacity of HRM to detect mixed infections.

Such infections are epidemiologically relevant, as they may facilitate genetic exchange, broaden host associations, and influence infection dynamics in peri-urban and sylvatic environments. Overall, the combined HRM–NGS workflow proved highly complementary, enabling rapid and cost-effective screening while providing accurate haplotype resolution even in samples with low DNA content. This integrative approach enhances genotyping reliability, reveals cryptic genetic diversity, and strengthens inference on transmission pathways between wild and domestic hosts, in line with current One Health surveillance recommendations.

### 4.5 One Health considerations

Although giardiasis in dogs and cats is predominantly associated with host-adapted assemblages, the detection of zoonotic assemblages in wildlife highlights the relevance of cross-species transmission processes, particularly in peri-urban environments. Environmental contamination through water runoff, shared recreational spaces, and close human-animal contact may facilitate indirect transmission pathways. In this context, hunting dogs represent important bridging hosts linking sylvatic transmission cycles with domestic settings.

These dynamics should be explicitly considered in risk assessment frameworks for companion animals and vulnerable human populations. Overall, the findings emphasize the complex ecology of *G. duodenalis* at the domestic-wildlife interface in Bosnia and Herzegovina and underscore the need for continued molecular surveillance. Targeted monitoring along hydrological corridors and within expanding golden jackal populations, combined with integrated One Health strategies focusing on sanitation, responsible pet ownership, and environmental monitoring, will be essential components of future risk mitigation efforts.

### 4.6 Study limitations

This study was conducted under several practical constraints that should be considered when interpreting the findings. Sampling relied on animals obtained through veterinary clinics, shelters, kennels, and hunter-derived or road-kill carcasses, resulting in a heterogeneous collection reflecting diverse health, ecological, and management contexts. Certain wild mesocarnivore species, including martens, wolves, and wildcats, were represented by relatively few individuals, limiting the scope for species-specific inference. In addition, the geographical distribution of samples was not fully uniform, and collections were conducted over multiple years without predefined seasonal stratification. Consequently, the study was not designed to resolve fine-scale spatial variation or seasonal dynamics in *G. duodenalis* occurrence.

Several methodological limitations should also be acknowledged. Postmortem sampling of wild carnivores may influence detection sensitivity due to DNA degradation or variation in intestinal content integrity. Quantitative measures of infection, such as cyst burden or semi-quantitative PCR indicators, were not assessed, precluding evaluation of infection intensity and shedding patterns. Although HRM genotyping was supported by MinION sequencing for selected samples, minor discrepancies in melting-curve interpretation remain a theoretical possibility. Finally, the cross-sectional design of the study does not allow conclusions regarding transmission direction, temporal dynamics, or long-term persistence of assemblages. Because not all qPCR-positive samples yielded sequence-grade amplicons, some host-associated assemblages, particularly in feline isolates, may be under-represented in the genotyped subset.

Within these limitations, the study provides a coherent molecular dataset for Bosnia and Herzegovina and establishes a foundation for future investigations incorporating longitudinal designs, spatially structured sampling, and integrated One Health–oriented approaches.

## 5 Conclusion

Domestic mesocarnivores, particularly cats and hunting dogs, were identified as key hosts contributing to the circulation of *G. duodenalis* in Bosnia and Herzegovina, whereas wild mesocarnivores exhibited low but epidemiologically meaningful detection rates. The detection of the zoonotic assemblage B in foxes and golden jackals, together with occasional mixed infections, indicates limited yet relevant cross-species transmission at the domestic–wildlife interface. Hunting dogs, due to their movement across sylvatic and peri-urban environments, may act as bridging hosts connecting transmission cycles in wild and domestic settings.

Taken together, these findings suggest that giardiasis in domestic carnivores in Bosnia and Herzegovina is shaped primarily by ecological exposure and management practices, while assemblage distribution reflects a combination of host adaptation and sporadic zoonotic spillover. This study provides the first molecular evidence of *G. duodenalis* assemblage circulation among domestic and wild mesocarnivores in the country and highlights the need for expanded, longitudinal surveillance within an integrated One Health framework.

## CRediT authorship contribution statement

**Azra Bačić:** Writing – review & editing, Writing – original draft, Methodology, Investigation, Formal analysis, Data curation, Conceptualization. **Naida Kapo:** Writing – review & editing, Writing – original draft, Methodology, Investigation, Formal analysis, Data curation. **Jasmin Omeragić:** Writing – review & editing, Methodology, Funding acquisition, Formal analysis, Conceptualization. **Šejla Goletić Imamović:** Writing – review & editing, Software, Methodology, Formal analysis. **Toni Eterović:** Writing – review & editing, Methodology, Investigation. **Ilma Terzić:** Writing – review & editing, Methodology, Investigation. **Adis Softić:** Writing – review & editing, Software, Methodology, Formal analysis. **Vedad Škapur:** Writing – review & editing, Investigation, Formal analysis. **Teufik Goletić:** Writing – review & editing, Writing – original draft, Validation, Supervision, Methodology, Funding acquisition, Formal analysis, Conceptualization.

## Ethical statement

This study was conducted in accordance with the Veterinary Law of Bosnia and Herzegovina (Official Journal of Bosnia and Herzegovina, no: 34/02) and the Animal Protection and Welfare Law in Bosnia and Herzegovina (Official Journal of Bosnia and Herzegovina, no: 25/2009 and 9/2018). It was approved by the Ethics Committee of the Veterinary Faculty, University of Sarajevo, Bosnia and Herzegovina (Approval no. 07-03-1137-2/24, approval date: January 23, 2025).

## Declaration of generative AI and AI-assisted technologies in the manuscript preparation process

During the preparation of this work the authors used ChatGPT, OpenAI in order to create Graphical abstract. After using this tool/service, the authors reviewed and edited the content as needed and takes full responsibility for the content of the published article.

## Funding

This work was supported by the Environmental Protection Fund of the Federation of Bosnia and Herzegovina (Grant No. 01-09-2-1581/2024), under the scientific project “Monitoring of zoonotic diseases of wild game in the area of Sarajevo Canton with the aim of preserving and improving biodiversity”. The funder had no role in study design; in the collection, analysis, and interpretation of data; in the writing of the manuscript; or in the decision to submit the article for publication.

## Conflict of interest

The authors declare no conflicts of interest.
